# Oligonucleotide Frequencies of Barcoding Loci Can Discriminate Species across Kingdoms

**DOI:** 10.1371/journal.pone.0012330

**Published:** 2010-08-20

**Authors:** Antariksh Tyagi, Sumit K. Bag, Virendra Shukla, Sribash Roy, Rakesh Tuli

**Affiliations:** Center for Plant Molecular Biology, National Botanical Research Institute (Council of Scientific and Industrial Research), Lucknow, India; GSF Research Center for Environment and Health, Germany

## Abstract

**Background:**

DNA barcoding refers to the use of short DNA sequences for rapid identification of species. Genetic distance or character attributes of a particular barcode locus discriminate the species. We report an efficient approach to analyze short sequence data for discrimination between species.

**Methodology and Principal Findings:**

A new approach, Oligonucleotide Frequency Range (OFR) of barcode loci for species discrimination is proposed. OFR of the loci that discriminates between species was characteristic of a species, i.e., the maxima and minima within a species did not overlap with that of other species. We compared the species resolution ability of different barcode loci using *p*-distance, Euclidean distance of oligonucleotide frequencies, nucleotide-character based approach and OFR method. The species resolution by OFR was either higher or comparable to the other methods. A short fragment of 126 bp of internal transcribed spacer region in ribosomal RNA gene was sufficient to discriminate a majority of the species using OFR.

**Conclusions/Significance:**

Oligonucleotide frequency range of a barcode locus can discriminate between species. Ability to discriminate species using very short DNA fragments may have wider applications in forensic and conservation studies.

## Introduction

The concept of DNA barcoding i.e. use of short DNA sequences for rapid identification of species is increasingly gaining support from conservationists and taxonomists [1]–[6]. In animals, the mitochondrial gene, *COI* has been widely recognized as a barcode [2], [4], [7]–[9]. In case of plants, The Plant Working Group of the Consortium for the Barcode of Life (CBOL) has recently proposed a two-locus combination of *matK* and *rbcL* as the standard plant barcode [1]. Several other candidate barcodes have also attracted the attention of many researchers [1], [5], [10]–[13]. CBOL is fostering the development of international research alliances to build a barcode library for all eukaryotic organisms. Central to the DNA barcoding is a database of previously identified reference specimens and their corresponding barcode loci sequences. Most of the DNA barcode literature describes species resolution either by genetic distance based approach or nucleotide-character based approach. Using distance based approach, accurate assignment of query sequence to particular taxa may be misleading when there is overlap of intra- and interspecific distances because of variable rates of evolution between and within species [14]–[16]. CBOL plant working group used non-overlapping intra- and interspecific nucleotide distances as a criterion for species discrimination in land plants [1]. An alternate to distance based approach is the character based barcoding [17]–[19]. In this approach, species are identified on the basis of the presence or absence of a particular diagnostic nucleotide(s), either singly (simple character) or in combination (compound characters). This approach is based on the assumption that members of a species share sequence attributes that are absent in a sister species [19].

We examined whether oligonucleotide frequencies in different barcode loci can discriminate species.In earlier studies, oligonucleotide frequencies have been reported to exhibit species specific signals [20]–[25], but most of these studies were based on the analysis of whole genome. Thus these were applied to small genomes only and used for classification of bacteria. Phylogenetic clustering was based on Euclidean distances derived from such oligonucleotide frequencies. No attempt was made to evaluate whether nucleotide frequencies in small regions of around 650 bp or less could distinguish species across eukaryotes. We describe a new method of non-overlapping oligonucleotide frequency ranges for species identification and compare its species resolution ability with *p*-distance, Euclidean distance (derived from oligonucleotide frequency) and nucleotide character based methods, using standard barcode loci. Species discrimination by this method can be performed using a program, Oligonucleotide Frequency Barcode Generator, developed by us, is freely available at http://www.nbri.res.in/ofbg/ofbg.aspx.

## Materials and Methods

### Nucleotide sequences

We used barcode loci sequences reported in different studies including those available in BOLD (Barcode of life Database, http://www.boldsystems.org) and NCBI GenBank. These included 2777 sequences for *COI* region for species ranging from fungi to mammals, 251 sequences of *matK*, 258 sequences of *rbcL* for land plants and 180 sequences of ITS for plants and fungi. Each group was represented by multiple genera and congeneric species with multiple accessions in each species. The *matK* and *rbcL* sequences were taken from CBOL plant working group (1). Few sequences were deleted from CBOL dataset due to short lengths. The details of the sequence data are given in Table 1.

**Table 1:** Details of sequences used in this study.

Locus	Database	Dataset	Number of genera	Number of species	Number of accessions	Average sequence length
*COI*	BOLD- CBAM	CO1 Barcoding Amphibians [37]	10	29	271	587.9
	BOLD- ACMC	Mosquitoes of North America [43]	10	48	271	585
	BOLD- PSP	Penicillium [44]	4	70	353	531
	BOLD- AROM	Royal Ontario Museum - Birds [45]	40	79	349	554
	BOLD- EWSHK	Sharks [28]	52	74	1030	650.1
	BOLD- ABSMS	Small Mammal Survey in Bakhuis, Suriname [46]	49	71	503	579
ITS	GenBank	Agaricus	1	17	48	638.5
	GenBank	Alexandrium	1	11	40	486.6
	GenBank	Ephedra	1	9	21	1660.7
	GenBank	Nymphaea	1	11	33	590.5
	GenBank	Oryza	1	12	34	590
*matK*	GenBank	Land plants [1]	30	83	215	644
*rbcL*	GenBank	Land plants [1]	33	96	258	507

The figures in parenthesis indicate the references from where sequences were taken.

### Oligonucleotide frequency

The nucleotide sequences of a particular locus were aligned using clustalW [26] implemented in MEGA4.0 [27]. The aligned sequences were trimmed off from both 5′ and 3′ ends to make datasets of equal aligned length. The alignment was then removed from this dataset and oligonulceotide frequencies were determined from this unaligned dataset. Di- or trinucleotide frequencies of a sequence was calculated by the occurrence of a particular di- or trinucleotide in a sequence divided by the total number of di- or trinucleotides i.e n-1 and n-2 respectively, where, n is the length of a particular sequence. The oligonucleotide occurrence was calculated using shift of single nucleotide window. After calculating the oligonucleotide frequencies of all sequences, the minimum and the maximum frequency of a particular di-or trinucleotide in a given species were calculated. If S_i,j_ represents, *j*th accesion of *i*th species, where, *i* varies from 2 to m and *j* varies from 2 to n, then
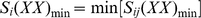


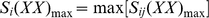
where, m and n are the number of species and accessions of a particular species, respectively. *XX* refers to a particular dinucleotide and *XXX* for a particular trinucleotide. For a particular species pair we considered at least two accessions per species to calculate the range of the minimum and the maximum oligonucleotide frequency. These values were used to generate a binomial matrix. The Euclidean distances (D) based on oligonucleotide frequency differences were calculated as follows.
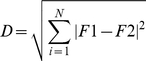
Where, *N* is the number of oligonucleotides, *F*1 and *F*2 represent the frequency of each type of oligonucleotide for species 1 and 2, respectively. Each distance was calculated from di- and tri-nucleotide frequencies.

### Species discrimination using oligonucleotide frequency, p-distance and nucleotide character based methods

We compared the range of the minimum and the maximum di- or trinucleotide frequencies between two species in all combinations. If the range of the minimum and the maximum di- or trinucleotide frequencies of a species did not overlap and were separated from each other by at least a value of 1/a-1 or 1/a-2 (threshold value, t) for di and trinucleotide respectively, where ‘a’ is the average sequence length, we considered the species as resolved. Thus in a binomial matrix, the species which were resolved among them were indicated by ‘1’ and those which were not resolved were indicated by ‘0’ (The matrix tables of datasets analyzed in this study are available on request). For species discrimination using *p*-distance, the sequences were aligned and pair wise uncorrected *p*-distances were calculated using MEGA4.0. Species resolution was considered as successful if the minimum interspecific *p*-distance involving a species was larger than its maximum intraspecific *p*-distance. Similarly, using Euclidean distance method, species recovery was considered successful if the minimum interspecific Euclidean distance derived from di- and trinucleotide frequencies was higher than the maximum intraspecific Euclidean distance involving a species. Species discrimination using character based method (simple pure and simple private characters), was determined following Rach *et al.* [18]. Wong *et al.* [28] used compound characters along with simple characters for species discrimination in sharks using 1030 *COI* sequences. We compared their results on species resolution with the OFR method.

### Statistical tests

Pearson's correlation coefficient test was applied between the differences of the minimum interspecific and the maximum intraspecific *p*-distances and i) differences of the minimum interspecific and the maximum intraspecific Euclidean distances ii) minimum number of non overlapping OFR's resolving the species. To identify the method that provides the highest species resolution amongst the six methods, the percentage species resolution data was normalized by arcsine transformation. Then, repeated measures ANOVA was applied with Newman-Keuls Post-Hoc test.

## Results

**Figure 1:**
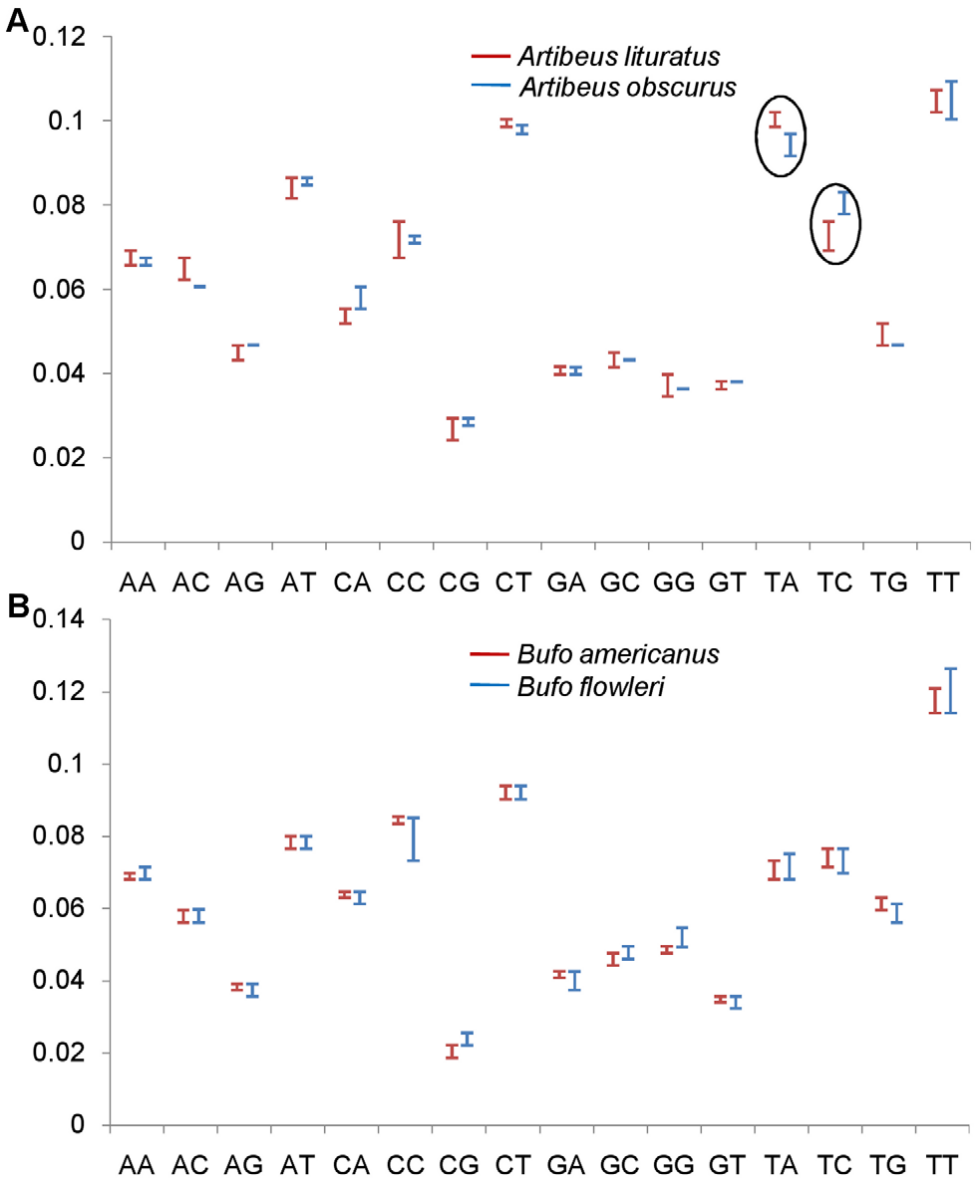
Resolution of species by dinulceotide frequency. In case of the species pair (*Artibeu slituratus* and *Artibeu sobscures*) which is resolved (A); the resolving dinucleotides (TA and TC; encircled) give non overlapping frequency range. The gap between the di-nucleotides in the two species is greater than the threshold values. The species pair (*Bufo americanus* and *Bufo floweri*) which is not resolved (B); shows overlap in all dinucleotide frequencies between the two species. X - axis, different dinucleotides and Y- axis, dinucleotide frequencies.

**Figure 2:**
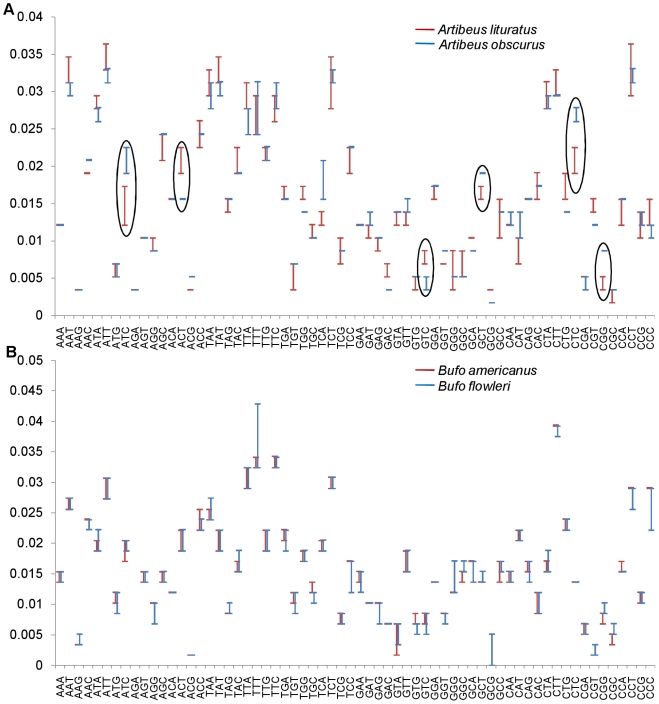
Resolution of species by trinulceotide frequency. In case of the species pair (*Artibeus lituratus* and *Artibeus obscures*) which is resolved (A); shows that in case of ATC, ACT etc. (encircled) do not show overlap in the frequency range. The species pair (*Bufo americanus* and *Bufo floweri*) which is not resolved (B); shows overlap in all trinucleocirclede frequencies between the two species. X - axis, different trinucleotides and Y- axis, trinucleotide frequencies.

The analyses showed that in case of species pairs resolved by using a barcode locus, the oligonucleotide frequency range of at least one oligonucleotide did not overlap with the frequency range of the same oligonucleotide for another species within a data set under study. For instance, the two species, *Artibeus lituratus* and *Artibeus obscures* were resolved by *COI* following distance based approach. The dinucleotide OFR's of TA and TC did not overlap in the two species (Figure 1A) and the gap between them was greater than the threshold values for differentiating the species pair. On the other hand, in case of *Bufo americanus* and *Bufo floweri* which were not resolved by *COI*, all the sixteen dinucleotide OFR's overlapped with each other (Figure 1B). Similarly, in case of trinucleotide frequency range, six trinucleotide OFR's did not overlap for *Artibeus lituratus* and *Artibeus obscures* species pairs (Figure 2A) and all the 64 OFRs overlapped for the *Bufo americanus* and *Bufo floweri* species pair (Figure 2B).

### Comparison between oligonucleotide frequency range, Euclidean, p-distance and nucleotide character based methods of species resolution

#### The animal and fungi barcode, *COI*

**Figure 3:**
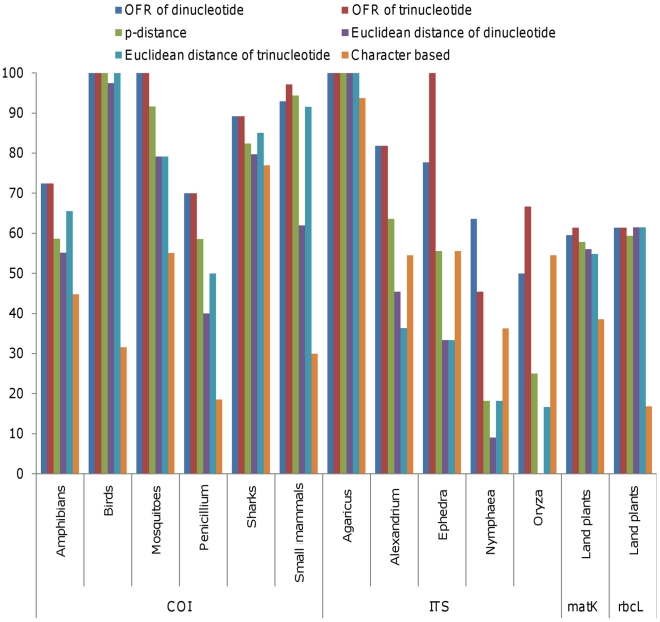
Percent species resolution by different methods using standard barcode loci. X - axis, species and barcode loci and Y- axis, percent species resolution.

The species discrimination ability of di- and tri nucleotide frequency was estimated from the binomial matrix table as described in materials and methods. The comparative species recovery by different barcode loci using *p*-distance, Euclidean distance, nucleotide character and OFR based methods is depicted in Figure 3. The species recovery using *COI* barcode locus differed in different groups of animals and fungi. In case of mosquitoes of North America, the species resolution by the *p*-distance, Euclidean distance and simple nucleotide character based methods was 91.6%, 79.1%and 55.1% respectively whereas the species resolution by di- and trinucleotide frequencies was 100%. Similarly, in amphibians, small mammals and *Penicillium*, species recovery by OFR was higher (72.4%, 97.0% and 70.0% respectively) than that by the *p*-distance (58.6%, 94.3% and 58.5% respectively), Euclidean distance (65.0%, 91.0%, and 50.0% respectively) and simple nucleotide character based methods (44.8%, 77.0% and 18.5% respectively). In case of birds, species recovery was 100% by applying *p*-distance, Euclidean distance and OFR methods where as simple nucleotide character based approach yielded only 31.6% species recovery. In sharks the species recovery using OFR method was higher (89.2%) than that by using compound character based approach (77.0%). Overall, the species resolution by di- and trinulceotide OFR was significantly higher than that by other methods and there was no significant difference in species resolution between di and trinucleotide OFR.

#### The plant barcodes, *matK* and *rbcL*

The *matK* and *rbcL* loci have widely been used as plant barcodes. We tested the species discrimination power of these loci, using oligonucleotide frequencies of the sequences reported by CBOL plant working group [1]. As evident from Figure 3, the species discrimination by OFR based method was higher than *p*-distance, Euclidean distance and nucleotide character based methods for both the loci.

#### The internal transcribed spacer region, ITS

The internal transcribed spacer regions are used in phylogenetic as well as in barcoding studies of organisms especially in fungi and plants. The species resolution by OFR was much higher than by the other three methods in all the groups.

#### Statistical tests

On the basis of Pearson's correlation coefficient test, the differences of the minimum interspecific and the maximum intraspecific *p*-distances are strongly correlated with the differences of the minimum interspecific and the maximum intraspecific Euclidean distances and also with the minimum number of non-over lapping OFR's (Table S1). In other words, the *p*-distance between sequences is in proportion with Euclidean distance and the number of non-overlapping OFR's. ANOVA was applied to identify the method that provides significantly the highest species resolution amongst different methods (Table S2). The percentage species resolution by OFR of trinucleotide and OFR of dinucleotide was significantly higher than the other methods. However, there was no significant difference in species resolution between the OFR method of di- and trinucleotide.

### Variation in sequence length and species recovery

**Figure 4:**
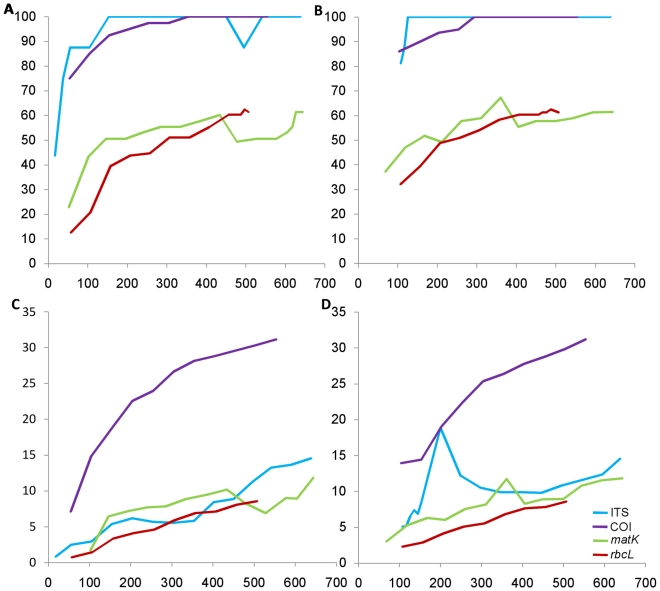
Percent species resolution (X-axis) with decrease in sequence length (Y-axis) from 5′ (A) and 3′ (B) ends and their corresponding average minimum number of differentiating trionucleotides (C) and (D).

We investigated the effect of length of a particular locus on species discrimination ability by oligo-nucleotide frequency. We chose the group which showed maximum species recovery for the locus concerned; for example, *COI* in birds. To find the shortest sequence length that gives species resolution equal to the full length sequence, we trimmed the sequences by steps of 50 bp from the 5′ and3′ ends one by one. For each reduced-length sequence set, species discrimination ability was calculated. The sequence length at which a drop in species resolution was observed, the trimming window size was reduced to 10 bp to get the finer minimum length for the maximum species recovery. As shown in Figure 4, the minimum average length at which maximum species recovery was obtained using *COI* for birds was 354 bp and 294 bp when sequences were trimmed off from5′ (Figure 4A) and 3′ (Figure 4B) ends respectively. For ITS locus, we examined the effect of trimming the ends on the resolution of species in the genus *Agaricus*. When sequences were trimmed off from 5′ (Figure 4A) and 3′ (Figure 4B) ends, the minimum average length of 153 and 126 bp respectively were able to resolve all the species in *Agaricus*. In case of *rbcL*, 457 bp and 407 bp were the minimum average lengths at which maximum species recovery were obtained when trimmed off from 5′ (Figure 4A) and 3′ (Figure 4B) ends respectively. However, in *matK*, there was no consistent trend of species resolution with decreasing length as observed in other loci. Overall, there is a gradual decrease in the number of oligonucleotides that differentiate a species pair, with corresponding decrease in sequence length (Figure 4C and 4D).

### Effect of indels on species resolution

Since the OFR method relies on average sequence length of a dataset, major deviation from the average length caused by indels may impact the overall species recovery. The minimum gap between the OFR's for separating a species is the threshold value, t (as described in materials and methods). Sequences shorter than the average sequence lengths due to deletions will be subjected to a weak threshold which may lead to false positives species resolution (Type I error). Similarly, sequences longer than the average length due to insertions will be subjected to a too stringent threshold which may lead to false negative species resolution (Type II error). The minimum length of an indel that can cause false positive or false negative results can be determined with the assumption that all the accessions in a species are of equal length. If the sequence length of the species pair under study is equal but varying from average sequence length, then the minimum length of an indel which can cause a Type I or Type II error can be determined by the following equation
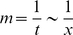



*m* = minimum length of indel which may lead to Type I or Type II error

*t* = threshold value for species resolution as described above

*x* = value of gap or overlap between OFR's of the oligonucleotide having the largest gap or overlap. If actual length of deletion in a species pair is larger than or equal to *m*, it may lead to a Type I error and if the actual length of insertion in a species pair is larger than or equal to *m*, it may lead to a Type II error.

Similarly, if the sequence length of the species pair under study is unequal due to indels, then, if the difference between the longer sequence (due to insertion) and the average sequence length is *D_l_* and the difference between the smaller sequence (due to deletion) and the average sequence length is *Ds*, then the following equation can be used to determine the Type I and Type II errors
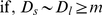
then if, D*_s_*>D*_l_*, it may lead to Type I error and if , D*_l_*>D*_s_*, it may lead to Type II error.

## Discussion

The three most important results of the present study are (i) the di- and trinucleotide frequencies of barcode loci can distinguish species, (ii) higher the nucleotide distance between a species pair, higher is the number of non-overlapping oligonucleotides between the two species and (iii) species discriminating ability of the OFR approach is higher than the commonly used approaches.

The species-specific signals for oligomers up to a length of four nucleotides have been identified [29], [30]. The oligonucleotide frequency is known to be different between species but consistent among the genes of a species [31]. More recently, Takashi *et al.* [32] analyzed bacterial species phylogeny, using oligonucleotide frequency distances. They suggested that oligonulceotide frequency is useful not only for classification of bacteria, but also for estimation of phylogenetic relationships among closely related species. This and other reports [20]–[25] considered the whole genome sequences for species clustering by Euclidean distance derived from oligonucleotide frequencies. Our study shows that the barcode loci can efficiently discriminate species using di- or trinucleotide frequencies of the loci across the kingdom. If the sequences are of full length genes or of equal length and the same region of a locus, the method does not require multiple alignments of the sequences. However, since the sequences available in the public data base are of variable lengths, we aligned the sequences to make a uniform length dataset after trimming from 5′ and 3′ ends. This eliminates the chances of false positives and false negatives due to variation in sequence length. Such errors can also be caused by indels. The impact of indels on species resolution can be determined by the equations as described in results.

CBOL plant working group recently reported that the minimum interspecific *p*-distance should be larger than the maximum intra- specific *p*-distance for a species to be considered as resolved. Our analysis is based on the range of the minimum and the maximum di- or trinucleotide frequencies of a barcode locus sequence for a particular species. This approach yielded higher species recovery than the distance based approach. This may be due to the fact that a single nucleotide substitution in a sequence causes change in number of di- and trinucleotides by 2 and 3 respectively. In distance based approach, species identification is based on the observation that intraspecific genetic divergence is usually lower than the inter-specific divergence [33]. Several reports provide an extensive explanation of why distances are inappropriate for species circumscription [16], [17], [34]–[36]. Others have advocated the genetic distance threshold based approach for species identification. For example in amphibians, a threshold of 5% for a fragment of the 16S rRNA gene and 10% for the *COI* gene has been suggested for species circumscription [37]. In threshold based approach for species identification, the major concerns are the variation in the rate to fix a threshold and that a single locus threshold can be confounded by introgression or selection [38], [39]. However, a biological species *sensu* or phylogenetic species *sensu* could, in theory, differ only by a single nucleotide change [40]. In our method a single nucleotide substitution is reflected in oligonucleotide frequencies between a species pair.

The OFR and Euclidean distances derived from oligonucleotide frequencies are highly correlated with the *p*-distances. This shows that the relative distances between species based on oligonucleotide frequencies of barcode loci are similar to that of *p*-distances. Therefore OFR's and Euclidean distances derived from oligonucleotide frequencies also indicate phylogenetic relationship between species. Further, to eliminate errors (Type I and II), we used the stringency of the minimum required gap, the threshold value, t for differentiating between species. These results suggest that oligonucleotide frequency based database of barcode sequences can be useful for rapid species identification in preference to the distance based barcoding database.

The length of a particular locus is important for barcoding, especially in case of degraded samples or herbarium and museum preserved specimens where quality sequence lengths are difficult to obtain. Our results show that smaller parts of barcode loci can be used to distinguish species by the OFR method. Further, as we reduce the length of a particular locus, the number of non-overlapping oligonucleotides that differentiate a species pair decreases; thus the confidence of species discrimination also decreases. The fact that in all the four loci, the maximum species recovery at minimum length was observed when sequences were trimmed off from 3′ end indicates that the nucleotide substitutions are more frequent at 5′ end than at the 3′ end of these sequences. By *in silico* approach, Hjibabaei *et al.* [41] reported that mini barcodes of 109 bp and 218 bp for fishes and Lepidoptera respectively, were as good as full length *COI* barcode. On the other hand, by deploying *COI* Meusner *et al.* [42] showed that while the full-length DNA barcodes perform best (97% species resolution), 90% identification success is obtained with 100 bp and 95% success with 250 bp barcodes. The differences in size of the mini barcode may be due to different species used and different approaches followed for species resolution by different authors.

There are a few shortcomings of the OFR method that have not escaped our attention. First, large indels in the sequences may give erroneous results. Secondly, OFRs are dependent on sample size of a species; addition of new samples may change the OFR of a species. However, these drawbacks are inherent to traditional barcoding approach also. Despite these limitations, our approach provides an efficient tool for species identification using an effective barcode locus.

## Supporting Information

Table S1 Pearson's correlation coefficient test between p-distances and other four methods.
(0.05 MB DOC)

Table S2 Post hoc analysis of repeated measures ANOVA between the species resolution by different methods.
(0.04 MB DOC)
