# Supplementary material for: Oligonucleotide Frequencies of Barcoding Loci Can Discriminate Species across Kingdoms
Source: PLoS One. 2010 Aug 20;5(8):e12330. doi: 10.1371/journal.pone.0012330 (PMC2924895; doi:10.1371/journal.pone.0012330)
Supplement: Table S1 — Pearson's correlation coefficient test between p-distances and other four methods. (0.05 MB DOC) [file pone.0012330.s001.doc]

Table S1: Pearson's correlation coefficient test between *p*-distances and other four methods

| Locus | Database | R [*p*-Distance, dinucleotide Euclidean distance] | R [*p*-Distance, trinucleotide Euclidean distance] | R [*p*-Distance,OFR of dinucleotide ] | R [*p*-Distance,OFR of trinucleotide ] |
| --- | --- | --- | --- | --- | --- |
| ITS | Nymphaea | 0.0732 (0.01)* | 0.910 (0.0001) | 0.461 (0.154) | 0.714 (0.014) |
|  | Ephedra | 0.912 (0.001) | 0.918 (0.0004) | 0.646 (0.060) | 0.663 (0.051) |
|  | Oryza | 0.917 (0.0001) | 0.0924 (0.001) | 0.803 (0.002) | 0.789 (0.002) |
|  | Alexandrium | 0.916 (0.0001) | 0.955 (0.0001) | 0.844 (0.001) | 0.808 (0.003) |
|  | Agaricus | 0.383 (0.129) | 0.769 (0.0003) | 0.420 (0.094) | 0.770 (0.0003) |
| *rbcL* | Land plants | 0.898 (0.0001) | 0.907 (0.0001) | 0.811 (0.0001) | 0.892 (0.0001) |
| *matK* | Land plants | 0.818 (0.0001) | 0.863 (0.0001) | 0.634 (0.0001) | 0.724 (0.0001) |
| *COI* | Penicillium | 0.905 (0.0001) | 0.912 (0.0001) | 0.761 (0.0001) | 0.818 (0.0001) |
|  | Sharks | 0.702(0.0001) | 0.857(0.0001) | 0.720(0.0001) | 0.807(0.0001) |
|  | Mosquitoes | 0.753 (0.0001) | 0.809 (0.0001) | 0.737 (0.0001) | 0.749 (0.0001) |
|  | Birds | 0.634 (0.0001) | 0.885 (0.0001) | 0.448 (0.0001) | 0.708 (0.0001) |
|  | Amphibians | 0.907 (0.0001) | 0.946(0.0001) | 0.720 (0.0001) | 0.879 (0.0001) |
|  | Small Mammals of Suriname | 0.746 (0.0001) | 0.842 (0.0001) | 0.678 (0.0001) | 0.780 (0.0001) |

* Figures in parenthesis indicate *p*-values of correlations
