# Supplementary material for: Oligonucleotide Frequencies of Barcoding Loci Can Discriminate Species across Kingdoms
Source: PLoS One. 2010 Aug 20;5(8):e12330. doi: 10.1371/journal.pone.0012330 (PMC2924895; doi:10.1371/journal.pone.0012330)
Supplement: Table S2 — Post hoc analysis of repeated measures ANOVA between the species resolution by different methods. (0.04 MB DOC) [file pone.0012330.s002.doc]

Table S2: Post hoc analysis of repeated measures ANOVA between the species resolution by different methods

| Overall *p*<0.0001 |  |  |  |
| --- | --- | --- | --- |
| Newman-Keuls Multiple Comparison Test | Mean Diff. | q | *p* value |
| Character based vs OFR of trinucleotide | -34.8 | 8.657 | *p* < 0.001 |
| Character based vs OFR of dinucleotide | -32.97 | 8.2 | *p*< 0.001 |
| Character based vs p-distance | -19.82 | 4.929 | *p* < 0.01 |
| Character based vs Euclidean distance of trinucleotide | -14.24 | 3.542 | *p*< 0.05 |
| Character based vs Euclidean distance of dinucleotide | -8.604 | 2.14 | *p*> 0.05 |
| Euclidean distance of dinucleotide vs OFR of trinucleotide | -26.2 | 6.517 | *p*< 0.001 |
| Euclidean distance of dinucleotide vs OFR of dinucleotide | -24.36 | 6.06 | *p*< 0.001 |
| Euclidean distance of dinucleotide vs p-distance | -11.21 | 2.789 | *p*> 0.05 |
| Euclidean distance of dinucleotide vseuclidean distance of trinucleotide | -5.639 | --- | *p* > 0.05 |
| Euclidean distance of trinucleotidevs OFR of trinucleotide | -20.56 | 5.114 | *p*< 0.01 |
| Euclidean distance of trinucleotidevs OFR of dinucleotide | -18.73 | 4.657 | *p*< 0.01 |
| Euclidean distance of trinucleotidevs p-distance | -5.575 | --- | *p*> 0.05 |
| *p*-distance vs OFR of trinucleotide | -14.99 | 3.728 | *p* < 0.05 |
| *p*-distance vs OFR of dinucleotide | -13.15 | 3.271 | *p*< 0.05 |
| OFR of dinucleotide vs OFR of trinucleotide | -1.837 | 0.4569 | *p*> 0.05 |
